# A ^1^H-NMR-Based Metabonomic Study on the Anti-Depressive Effect of the Total Alkaloid of Corydalis Rhizoma

**DOI:** 10.3390/molecules200610047

**Published:** 2015-05-29

**Authors:** Hongwei Wu, Peng Wang, Mengting Liu, Liying Tang, Jing Fang, Ye Zhao, Yi Zhang, Defeng Li, Haiyu Xu, Hongjun Yang

**Affiliations:** Institute of Chinese Materia Medica, China Academy of Chinese Medical Sciences, Dong Nei Nan Xiao Jie 16, Beijing 100700, China

**Keywords:** total alkaloids of YuanHu (YHTA), corydalis rhizoma, metabonomics, ^1^H-NMR, depression, plasma, OPLS-DA

## Abstract

Corydalis Rhizoma, named YuanHu in China, is the dried tuber of Corydalis *yanhusuo* W.T. Wang which is used in Traditional Chinese Medicine for pain relief and blood activation. Previous pharmacological studies showed that apart from analgesics, the alkaloids from YuanHu may be useful in the therapy of depression by acting on the GABA, dopamine and benzodiazepine receptors. In this study, the antidepressive effect of the total alkaloid of YuanHu (YHTA) was investigated in a chronic unpredictable mild stress (CUMS) rat model using ^1^H-NMR-based metabonomics. Plasma metabolic profiles were analyzed and multivariate data analysis was applied to discover the metabolic biomarkers in CUMS rats. Thirteen biomarkers of CUMS-introduced depression were identified, which are myo-inositol, glycerol, glycine, creatine, glutamine, glutamate, β-glucose, α-glucose, acetoacetate, 3-hydroxybutyrate, leucine and unsaturated lipids (L7, L9). Moreover, a metabolic network of the potential biomarkers in plasma perturbed by CUMS was detected. After YHTA treatment, clear separation between the model group and YHTA-treated group was achieved. The levels of all the abnormal metabolites mentioned above showed a tendency of restoration to normal levels. The results demonstrated the therapeutic efficacy of YHTA against depression and suggested that NMR-based metabolomics can provide a simple and easy tool for the evaluation of herbal therapeutics.

## 1. Introduction

Depression is a serious mental disease. The mechanisms of depression are quite complex. As a popular accepted hypothesis of depression pathogenesis, the monoamine hypothesis suggests that depression results from an imbalance in neurotransmitters such as norepinephrine and dopamine [1]. In the clinic, although synthetic chemical anti-depressive drugs have proven effect, high toxicity and side effects such as somnolence and hepatotoxicityare very common [2,3]. In the search for new antidepressive drugs with good therapeutic effects and low toxicity, Traditional Chinese Medicine (TCM) and other ethnopharmacologies have attracted considerable attention. Compared with single target-based modern pharmacology, the most essential features of TCM are the use of multiple components for multiple targets and the synergistic therapeutics. However the scientific evidence about the efficacy and pharmacological mechanism(s) of TCM need further investigation.

Corydalis Rhizoma, known as YuanHu in China, is a famous herbal drug which is prepared from the dried tuber of *Corydalis yanhusuo* W.T. Wang. In TCM, YuanHu is believed to have the function of activating blood, moving ‘Qi’ (vital energy), and relieving pain [4]. To date, the active chemical constituents of YuanHu have been isolated and identified as tertiary and quaternary alkaloids which are responsible for the biological activities of the crude drug [5]. Previous research showed that alkaloids from YuanHu such as tetrahydropalmatine exert their pharmaceutical effect by interfering with neurotransmitters in the central nervous system corresponding to dopamine receptors, 5-hydroxytryptamine serotonin receptor, α-adrenergic receptor and GABA-A receptor, *etc.* [6,7,8,9]. Our previous study revealed that alkaloids of YuanHu also had the antidepressant and antianxiety pharmacologic actions. The alkaloids from *Corydalis* such as corydaline, tetrahydroberberine and protopine were connected with GABA receptors, dopamine receptor, and benzodiazepine receptor by a computational drug-target network, which assumed that alkaloids of *Corydalis* might be a source of potential anti-depressive drugs. In addition, the anti-depression efficacy was preliminarilyvalidated by the forced swimming test (FST) and the tail suspension test (TST) [10].

Metabolomics, a systemic assessment for the comprehensive and quantitative analysis of the changes in global metabolites in a biological matrix, has been increasingly used as a versatile tool for the discovery of molecular biomarkers for exploring the potential mechanisms of diverse diseases and assessing the therapeutic effects of drugs [11,12,13]. Metabolomics has recently attracted much attention in TCM research [14,15]. Among the analytical techniques used in metabolomic studies, proton (^1^H)-nuclear magnetic resonance (NMR) is a non-invasive, non-biased and easily quantifiable method [16,17]. It can measure various biofluids such as urine, plasma and cerebrospinal fluid directly without chemical derivatization or complicated sample preprocessing. ^1^H-NMR spectroscopy of biofluids is capable of simultaneously detecting a variety of low-molecular-weight metabolites and providing a wide ranging metabolic fingerprint of the analyzed biofluids. ^1^H-NMR spectra combined with multivariate statistical analysis, such as principal component analysis (PCA), partial least squares discriminant analysis (PLS-DA), and orthogonal partial least-squares discriminant analysis (OPLS-DA), has been widely used in metabolic profiling studies [18,19].

In this study, to confirm the antidepressant-like effect of alkaloids from YuanHu and explore the possible pharmacological mechanism behind the physiological and behavioral outcomes such as body weight changes, restlessness, torpor or lack of eating were measured in the chronic unpredictable mild stress (CUMS) rat model. The CUMS model is a well-validated and widely used rodent model of depression [20,21]. In CUMS, animals are exposed to a sequential series of relatively mild stressors that mimic the stresses in human life [22,23]. A ^1^H-NMR-based metabolomic strategy was then applied to evaluate the systemic metabolic consequences in CUMS model rats. Further, the effect of total alkaloid of YuanHu (YHTA) intervention in CUMS rats was evaluated. Multivariate statistical analysis of PCA, PLS-DA and OPLS-DA were applied to classify and interpret the obtained metabolic datasets with or without drug treatment. The characteristics of physiological metabolite levels in this rat model of CMUS and the antidepressive mechanism of YHTA were investigated.

## 2. Results and Discussion

### 2.1. Effect of YHTA on Behavioral Tests

**Figure 1:**
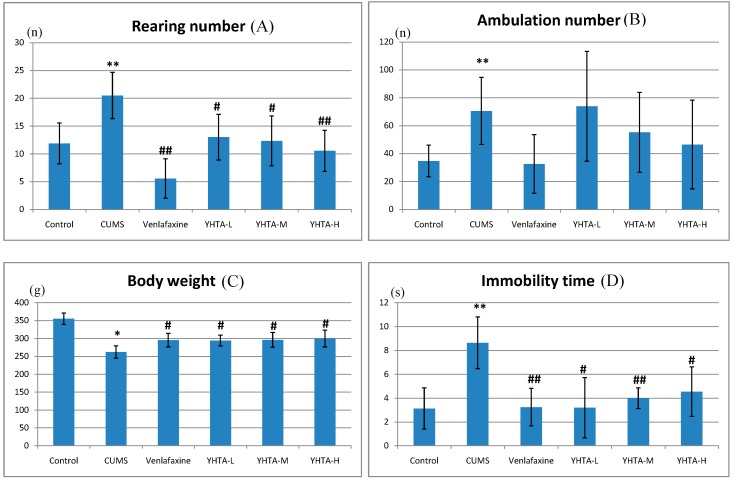

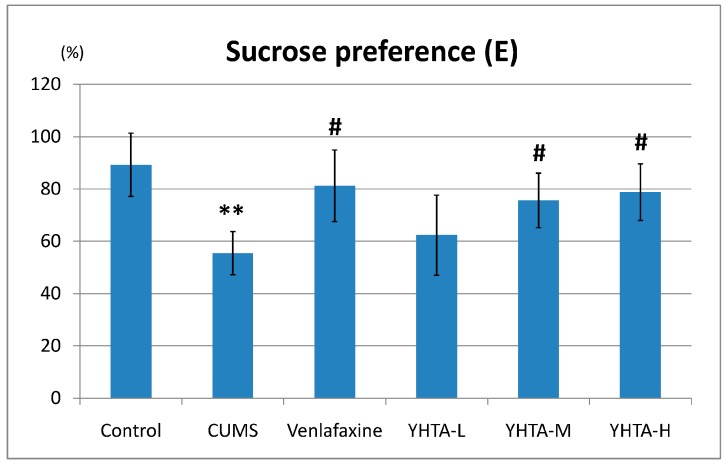
Effect of YHTA and venlafaxineon behavioralchanges in rats exposed to CUMS on the 21th day (mean ± SD, *n* = 8). Control: control group; CUMS: CUMS model group; Venlafaxine: venlafaxine group; YHTA-H: High dose group of YHTA; YHTA-M: Middle dose group of YHTA; YHTA-L: Low dose group of YHTA. * *p* < 0.05 *vs.* control group, ** *p* < 0.01 *vs.* control group, # *p* < 0.05 *vs.* model group, ## *p* < 0.01 *vs.* model group.

Open-field test, sucrose preference test and body weight were measured during the experimental period. As shown in Figure 1, on the 21th day, compared with the control group, the CUMS rats showed a significant increase in rearing number (A), ambulation number (B), immobility time (D) and a decrease in body weight (C) and sucrose preference (E). The behavior results indicated that a depressive-like behavioral status was obviously developed after 21-day CUMS exposure.

At the same time, these changes can be affected by the positive control drug and YHTA. Although there was no obvious effect of YHTA on the ambulation number, 21 days of YHTA administration at doses of 3, 6 and 12 g∙kg^−1^∙d^−1^ could significantly increase the body weight and decrease immobility time and rearing number of CUMS rats. Furthermore, treatment with middle and high dosages of YHTA could significantly increase the sucrose preference compared with that of CUMS rats. The results showed that YHTA-treated stressed groups had a tendency to return to the normal status.

### 2.2. Analysis of Plasma Samples by ^1^H-NMR Spectroscopy

**Figure 2:**
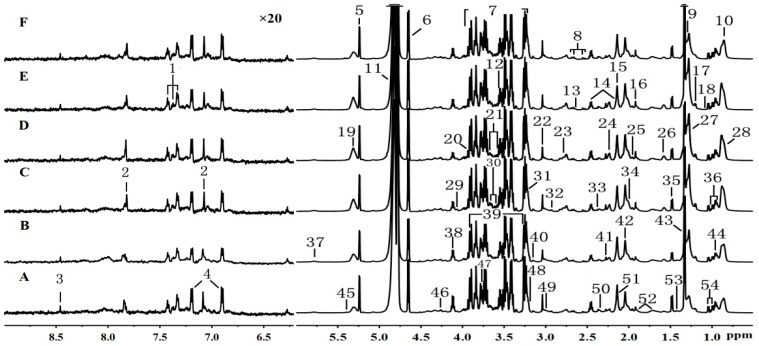
Representative ^1^H-NMR spectra (δ 0.5–6.0 and δ 6.2–9.0) of plasma obtained from groups **A**, **B**, **C**, **D**, **E** and **F**, which refer to the blank control group, model group, positive drug group, low dose group, middle dose group, high dose group, respectively. The region of δ6.2–9.0 (on the left side) was magnified 20 times compared with corresponding region of δ0.5–6.0 for the purpose of clarity. Keys: 1: phenylalanine; 2: histidine; 3: formate; 4: tyrosine; 5: α-glucose; 6: β-glucose; 7: α- & β-glucose; 8: citrate; 9: VLDL (L4), CH_3_-(CH_2_)_n_-; 10: VLDL (L2), CH_3_-(CH_2_)_n_-; 11: HOD; 12: glycine; 13,15: methionine; 14: glutamine; 16: acetate; 17: 3-hydroxybutyrate (3-HB); 18: isobutyrate; 19: lipid (L10), -CH=CH-; 20, 22: creatine; 21: glycerol of lipids; 23: lipid (L9), =CH-CH_2_-CH=; 24: lipid (L8), -CH_2_-C=O; 25: lipid (L6), -CH_2_-CH_2_-CH=CH-; 26: VLDL (L5), -CH_2_-CH_2_-C=O; 27: LDL (L3), CH_3_-(CH_2_)_n_-; 28: LDL (L1), CH_3_-(CH_2_)_n_-; 29, 30: *myo*-inositol; 31: phosphor-choline/glycerol-phosphocholine; 32: *N*,*N*-dimethylglycine; 33: pyruvate; 34: lipid (L7), ‑CH_2_-CH=CH-; 35: alanine; 36: isoleucine; 37: urea; 38, 43: lactate; 39: betaine; 40: malonate; 41: acetoacetate; 42: *N*-acetylglycoprotein signals; 44: leucine; 45: allanotin; 46: threonine; 47, 49, 52, 53: lysine; 48: cholesterol; 50, 51: glutamate; 54: valine.

Representative ^1^H-NMR spectra of the plasma from the blank control group, CUMS model group, venlafaxine-treated group and YHTA-treated group are shown in Figure 2. The ^1^H-NMR signals of all common low-molecular metabolites, such as aminoacids, organic acids and carbohydrates, were assigned according to previous publications [24,25] and public accessible metabolomic databases, such as HMDB and METLIN. The most intense signals were identified as lactate, alanine, glycine, methionine, glucose and *N*-acetylglycoprotein (NAG). Metabolites with the lower abundance were also detected and investigated. The region of δ 6.2–9.0 was magnified 20 times compared with corresponding region of δ 0.5–6.0 for the purpose of clarity (Figure 2). The useful extracted information was subsequently analyzed by multivariate statistics methods, including PCA, PLS-DA and OPLS-DA.

### 2.3. Multivariate Statistical Analysis of Plasma NMR Data of All Groups

**Figure 3:**
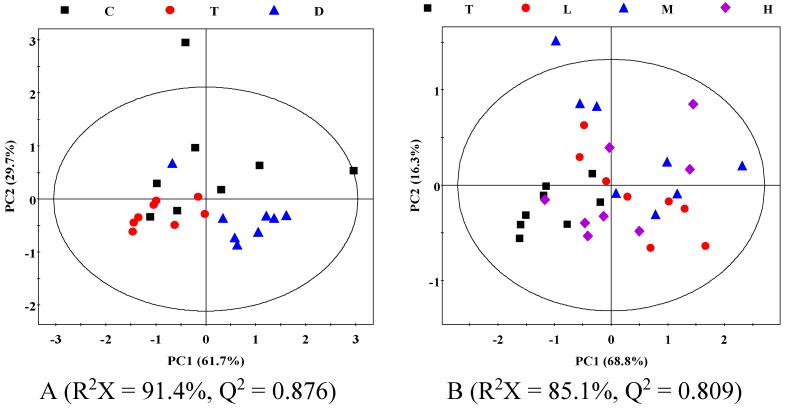
PCA scores plot based on NMR spectra of plasma obtained from different groups. C: Control group; T: CUMS Model group; D: positive drug; L: Low dose group of YHTA; M: Middle dose group of YHTA; H: High dose group of YHTA.

First, we analyzed the plasma metabolic profiles using unsupervised PCA. Although some degree of overlap was noted, the plot of PC1 *vs.* PC2 scores plot (Figure 3, R^2^X = 91.4%, Q^2^ = 0.876; R^2^X = 85.1%, Q^2^ = 0.809) showed a separation between the groups. This result mirrored the differences between the groups in the behavioral tests. These data demonstrated that the CUMS model was reliable and YHTA has an effect on the CUMS model.

**Figure 4:**
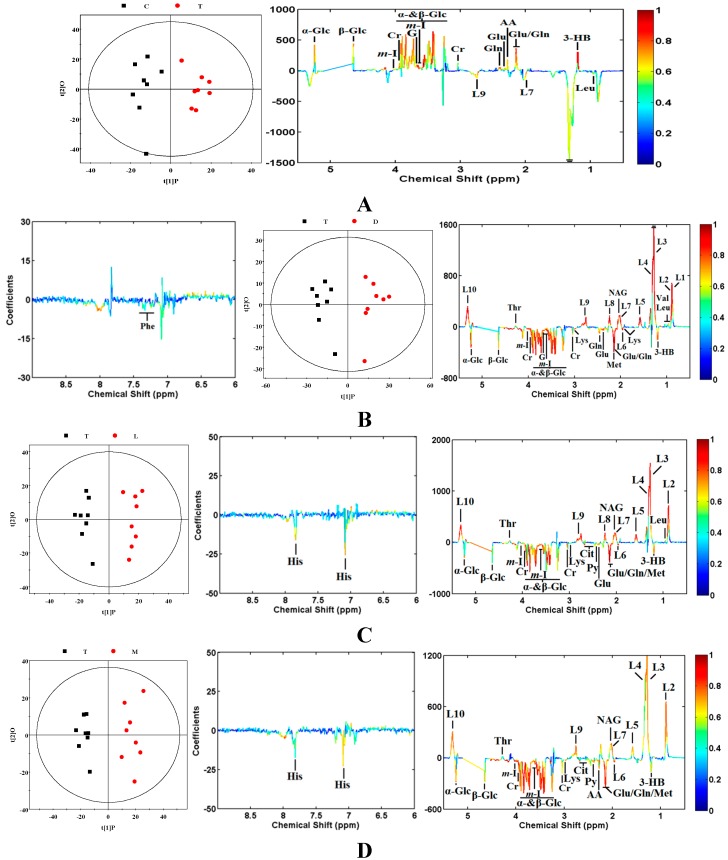

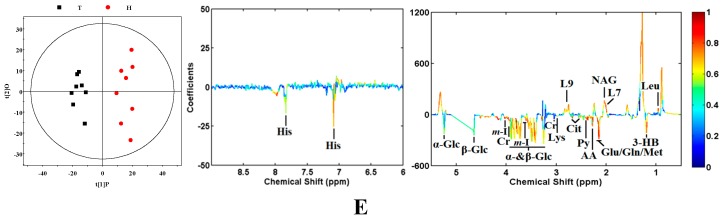
OPLS-DA scores plots (left panel) derived from ^1^H-NMR spectra of plasma and corresponding coefficient loading plots (middle and right panel) obtained from different dosage groups. The color maps show the significance of metabolites variations between the two classes. **A**. Control (C) *vs.* Model (T); **B**. Model (T) *vs.* Positive drug (D); **C**. Model (T) *vs.* Low dose group of YHTA(L); **D**. Mode (T) *vs.* Middle dose group of YHTA (M);**E**. Model (T) *vs.* High dose group of YHTA (H).

After an overview of the NMR data using PCA analysis, supervised analysis techniques such as PLS-DA and OPLS-DAwere then used, which can maximize the difference among the groups and aid in the screening of the marker metabolites responsible for class separation by removing systematic variations unrelated to pathological status. By applying PLS-DA, a 20-fold cross-validation was employed to obtain Q^2^ and R^2^ values, which represent the predictive ability of the model and the explained variance, respectively. To further validate the quality of the PLS-DA model, permutation tests consisting of a randomly permuting class membership and running 200 iterations were carried out. No over-fitting was observed according to the chance permutation results. Then a series of pairwise of OPLS-DA models were constructed subsequent to the PLS-DA analysis. OPLS-DA was performed to minimize the possible contribution of intergroup variability and to further improve the separation between the two groups. Characteristics of all models generated are summarized in Table 1, with acceptable goodness of fit, R^2^, and high-quality goodness of predication, Q^2^.As shown in the OPLS-DA scores plots (Figure 4), good separation between the groups was observed. Using a back-scaling transformation and projection to aid biomarker visualization, metabolites significantly contributing to the separation were clearly shown in the OPLS-DA coefficient loading plots (Figure 4). In these loading plots, positive peaks relative to zero represented levels of metabolites increased in the group which was in the positive direction of first principal component, whereas negative peaks indicated that levels of metabolites increased in the group which was in the negative direction of first principal component.

The CUMS rat biomarkers compared with the control group were confirmed by the coefficient loading plots (Figure 4A). The coefficient loading plot revealed that the 13 variables responsible for separation were those corresponding to *myo*-inositol, glycerol, glycine, creatine, glutamine, glutamate, β-glucose, α-glucose, acetoacetate, 3-hydroxybutyrate, leucine, and unsaturated lipids (L7, L9). Compared with the control group, increased levels of *myo*-inositol, glycerol, glycine, creatine, glutamine, glutamate, β-glucose, α-glucose, acetoacetate, and 3-hydroxybutyrate were found in CUMS rats. Leucine and unsaturated lipids (L7, L9) were significantly decreased in CUMS rats. These findings suggested that plasma metabolic pattern was significantly changed by the CUMS treatment.

**Table 1:** PLS-DA and OPLS-DA model summary for the discrimination between the groupsfrom NMR data.

	PLS-DA			OPLS-DA		
	R^2^X	R^2^Y	Q^2^	R^2^X	R^2^Y	Q^2^
C *vs.* T	39.0%	0.908	0.560	38.0%	0.908	0.521
T *vs.* D	42.8%	0.909	0.626	42.8%	0.909	0.615
T *vs.* L	37.1%	0.955	0.729	37.1%	0.955	0.684
T *vs.* M	41.7%	0.941	0.748	41.7%	0.941	0.678
T *vs.* H	35.1%	0.953	0.521	35.1%	0.953	0.515

C: Control group; T: CUMS Model group; D: positive drug group; L: Low dose group of YHTA; M: Middle dose group of YHTA; H: High dose group of YHTA.

From the OPLS-DA scores plot (Figure 4B–E), a significant biochemical distinction between the CUMS model group and YHTA-treated group was evident, as well as between the positive group and model group. From the OPLS-DA coefficient loading plots (Figure 4) and Table 2, we can see that except for the leucine level of the middle dose of YHTA and glycerol in the high dose of YHTA group, the 13 biomarkers of the CUMS model group in the drug treated groups all presented a tendency to return to normal status after YHTA and venlafaxine treatment. These results suggested that YHTA has a therapeutic effect in adjusting the changed levels of metabolites in CUMS rats. Moreover, the common and characteristic metabolites for the antidepressant effects of YHTA and venlafaxine revealed that some common and different metabolic pathways might be involved in the therapeutic mechanism. Exploration of the anti-depression mechanism of YHTA will be part of future studies in this project.

**Table 2:** Significantly changed metabolites in the different groups of rats exposed to YHTA from NMR data.

Metabolites	δ1H (ppm) and Multiplicity ^b^	R ^a^				
		C-T	T-D	T-L	T-M	T-H
Valine	0.99(d), 1.04(d)	-	↑0.695	-	-	-
Threonine	1.33(d), 3.58(d) 4.26(m)	-	↑0.810	↑0.788	↑0.683	-
Pyruvate	2.37(s)	-	↓−0.760	↓−0.694	↓−0.705	↓−0.871
Phenylalanine	7.33(d), 7.37(m) 7.42(dd)	-	↓−0.669	-	-	-
*N*-Acetylglycoprotein	2.04(s)	-	↑0.865	↑0.812	↑0.731	↑0.696
*myo*-Inositol	3.27(t), 3.56(dd), 3.62(t), 4.06(t)	↓0.851	↓−0.866	↓−0.906	↓−0.891	↓−0.863
Methionine	2.14(s), 2.65(t)	-	↓−0.927	↓−0.898	↓−0.795	↓−0.720
Lysine	1.72(m), 1.91(m), 3.01(m), 3.76(t)	-	↓−0.857	↓−0.806	↓−0.855	↓−0.735
Leucine	0.96(t)	↑−0.687	↑0.798	↑0.765	-	↑0.859
Lactate	1.33(d), 4.11(q)	-	↓−0.701	-	-	-
L9: Lipid, =CH-CH2-CH=	2.78(br)	↑−0.801	↑0.892	↑0.852	↑0.741	↑0.677
L8: Lipid, -CH2-C=O	2.23(br)	-	↑0.891	↑0.791	-	-
L7: Lipid,-CH2-CH=CH-	2.02(br)	↑−0.727	↑0.872	↑0.822	↑0.793	↑0.716
L6: Lipid, -CH2-CH2-CH=CH-	1.96(br)	-	↓−0.913	↓−0.862	↓−0.795	-
L5: Lipid, -CH2-CH2-C=O(VLDL)	1.56(br)	-	↑0.910	↑0.811	↑0.674	-
L4: Lipid, CH3-(CH2)n-(VLDL)	1.30(br)	-	↑0.885	↑0.799	↑0.718	-
L3: Lipid, CH3-(CH2)n-(LDL)	1.28(br)	-	↑0.866	↑0.782	↑0.696	-
L2: Lipid, CH3-(CH2)n-(VLDL)	0.90(br)	-	↑0.874	↑0.780	↑0.767	-
L10: Lipid, -CH=CH-	5.31(br)	-	↑0.865	↑0.796	↑0.725	-
Histidine	7.08(s), 7.84(s)	-	-	↓−0.856	↓−0.723	↓−0.683
Glycerol	3.57(m), 3.66(m), 3.78(m)	↓0.768	↓−0.785	↓−0.720	↓−0.684	-
Glycine	3.56(s)	↓0.802	↓−0.883	↓−0.770	↓−0.892	↓−0.738
Glutamate	2.10(m), 2.35(m), 3.78(t)	↓0.697	↓−0.910	↓−0.912	↓−0.823	↓−0.781
Glutamine	2.14(m), 2.45(m), 3.78(t)	↓0.669	↓−0.884	↓−0.817	↓−0.798	↓−0.745
Creatine	3.04(s), 3.93(s)	↓0.688	↓−0.846	↓−0.884	↓−0.821	↓−0.747
Citrate	2.53(d), 2.68(d)	-	-	↓−0.825	↓−0.764	↓−0.769
β-Glucose	3.25(dd), 3.41(t), 3.46(m), 3.49(t), 3.90(dd), 4.65(d)	↓0.792	↓−0.892	↓−0.872	↓−0.896	↓−0.774
α-Glucose	3.42(t), 3.54(dd), 3.71(t), 3.73(m), 3.84(m), 5.23(d)	↓0.799	↓−0.902	↓−0.915	↓−0.856	↓−0.770
Acetoacetate	2.28(s)	↓0.680	-	↓−0.673	↓−0.786	↓−0.779
3-Hydroxybutyrate	1.20(d), 2.31(dd), 2.41(dd)	↓0.776	↓−0.769	↓−0.711	↓−0.743	↓−0.790

R ^a^: Correlation coefficients, positive and negative signs indicate positive and negative correlation in the concentrations, respectively. The correlation coefficient of |r| > 0.666 was used as the cutoff value for the statistical significance based on the discrimination significance at the level of *p* = 0.05 and df (degree of freedom) = 7. ‘‘−’’ means the correlation coefficient |r| is less than 0.666; Multiplicity ^b^: s, singlet; d, doublet; t, triplet; q, quartet; dd, doublet of doublets; m, multiplet; br, broad single peak. C: control group; T: model group; D: positive drug; L: Low dose group; M: Middle dose group; H: High dose group. ↓ or ↑ represent the metabolite increased or decreased as compared with the model group.

### 2.4. Discussion

**Figure 5:**
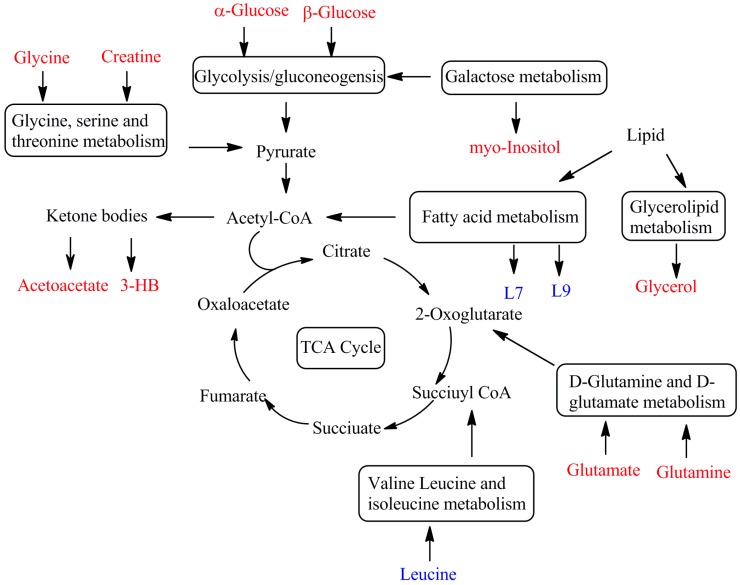
Metabolic network of the potential biomarkers in plasma that changed between the CUMS rats and normal controls. Significantly increased metabolites in the CUMS group are shown in red. Significantly decreased metabolites in the CUMS group are shown in blue. Levels of the potential biomarkers had a tendency to recover after intervention on CUMS treated rats with YHTA.

The plasma biomarkers of CUMS rats and YHTA-treated groups are involved in many metabolic pathways and physiological functions. The metabolic network of the 13 potential biomarkers in plasma that changed between the CUMS rats and normal controls is presented in Figure 5, which gives an overview of the metabolic pathways related to CUMS-induced depression. Among the 13 CUMS rat biomarkers glycine, glutamine, glucose, unsaturated lipids (L7, L9) and 3-hydroxybutyrate have been reported and their change tendencies were consistent to the previous reports [26,27,28,29], while the other biomarkers are reported for the first time in this study.

#### 2.4.1. Amino Acid Metabolism

Glycine, derived from serine, is an inhibitory neurotransmitter in the central nervous system [30]. Glutamate is the most abundant excitatory neurotransmitter in the vertebrate nervous system [31]. Glutamine, synthesized by the enzyme glutamine synthetase from glutamate and ammonia, is the most abundant amino acid found in blood plasma [32,33]. Compared with the control group, glycine, glutamate and glutamine were all increased in the CUMS group, which suggested that the biosynthesis of glycine, glutamate and glutamine was affected after CUMS treatment. These results were in agreement with a previous report on plasma metabolomic analysis of CUMS rats based on GC-MS [27]. After YHTA and venlafaxine treatment, glycine, glutamate and glutamine levels all show a tendency to return to normal status.

In addition, compared with the control group, leucine was significantly decreased in the CUMS group. As an essential amino acid, leucine cannot be synthesized by animals. Leucine is the only dietary amino acid that has the capacity to stimulate muscle protein synthesis [34]. It was believed that the decreased content of leucine was related with the weak diet under the physical stressors which caused the body weights losses of CUMS rats.

#### 2.4.2. Glycometabolism

Increased levels of β-glucose and α-glucose in blood were observed in the CUMS group. Glycometabolism has been suggested as a factor in depression studies. It was reported that patients with major depression have impaired insulin sensitivity as well as impaired oral glucose tolerance [35,36]. Depression occurs frequently in patients with diabetes mellitus. Glucose metabolism may be affected by the abnormal secretion of depression-related hormones. Dopamine and norepinephrine influences in the animal model appear to be hyperglycemic [37], and there are reports suggesting that antidepressant treatment is assumed to improve impaired glucose tolerance [38].

In addition, the level of *myo*-inositol, as a galatose metabolism product, in the blood of CUMS rats was found to be significantly increased compared with that in control group. There is study reporting that a significant reduction in *myo*-inositol was observed in depressive patients [39].

After YHTA intervention, the results showed a tendency of bringing the levels of β-glucose, α-glucose and *myo*-inositol back to normal, which suggests that YHTA can adjust the glycometabolic disorder caused by depression. There is also evidence showing that berberine, as the main alkaloid of *Corydalis*, has proven anti-diabetic effect by lowering blood glucose and regulating lipid metabolism [40,41,42].

#### 2.4.3. Lipid Metabolism and Energy Metabolism

Considerably increased levels of glycerol and decreased levels of unsaturated lipids (L7, L9) were observed in the CUMS group, suggesting a dysfunction of lipid metabolism. Acetoacetate and β-hydroxybutyrate, as endogenous ketone bodies, are produced by the liver from fatty acids for the body to use as energy during periods of low food intake (fasting) or in some pathological state such as diabetes mellitus [43]. Diabetes mellitus, hunger or acute alcohol poisoning can increase the content of acetoacetate and β-hydroxybutyrate in the blood. In this study, acetoacetate and β-hydroxybutyrate were observed to be increased significantly in the CUMS group compared with the levels in the control group, which suggested the dysfunction of the energy metabolism in CUMS rats.

Creatine serves an important protector of mitochondrial energy metabolism [44]. It was reported that creatine plays a pivotal role in brain energy homeostasis which is involved in the pathophysiology of depression [45]. In this study creatine were higher considerably in the CUMS group compared with the level in the control group.

In summary, after YHTA and venlafaxine administration, the level of the 13 biomarkers of CUMS rats mentioned above showed a tendency to return to normal levels. These results suggest that YHTA had an anti-depression effect by adjusting the amino acid metabolism, glycometabolism, lipid metabolism and energy metabolism. The common regulated biomarkers indicated that YHTA and venlafaxine may have some similar mechanisms, while YHTA intervention also influences some other endogenous metabolites, indicating that YHTA has other mechanisms. Further investigations are needed to fully elucidate the anti-depression mechanism of YHTA. The ^1^H-NMR-based metabolomics method used in this study provided a valid tool for the study of depression and herbal therapeutic evaluation.

## 3. Experimental Section

### 3.1. Chemicals and Reagents

Tubers of *C. yanhusuo* W. T. Wang were purchased from Dongjiaying Country, Hanzhong City, Shanxi Province, China, and identified by Professor Meihong Fu, Institute of Chinese Materia Medica, China Academy of Chinese Medical Sciences. Venlafaxine hcl sustained release capsules produced by Wyeth Medica Ireland (Little Connell, Newbridge, Co Kildare, Ireland) were bought from the Jin Xiang pharmacy (Beijing, China). D_2_O was purchased from Sea Sky Bio Technology Co. Ltd (Beijing, China). All other chemical reagents were analytical grade.

### 3.2. Preparation of YHTA

The total alkaloids of YuanHu (YHTA) were prepared as follows: briefly, 1kg of dried *Corydalis yanhusuo* W. T. Wang tuber was powdered and extracted with 95% ethanol (5 L) by hot reflux extraction for 1 h, a process that was repeated twice. After filtration, the extracts were combined and concentrated by vacuum evaporation at 40 °C until no alcohol odor was noticeable. Then the extract was dissolved in water (100 mL) and purified with 500 g of weak polar macroporous resin (AB-8). Initially the resin column was washed with five body volumes (BV) of 10% ethanol to remove impurities. Then 6 BV of 50% ethanol was used to obtain the total alkaloids of YuanHu. The YHTA eluent was dried *in vacuo* (40 °C) and ground to a powder for use. The yield of the extraction was about 1.5%. The powder was dissolved into water at three different concentrations for use. The total alkaloid content of YHTA was determined according to the acid dye colorimetry method using UV spectrophotometry with tetrahydropalmatine as control [46]. The total alkaloid content of YHTA was expressed as tetrahydropalmatine equivalents (g TE/g) and the result was 0.45 (g TE/g).

### 3.3. Animals

Forty-eight Sprague–Dawley rats weighing 140–150 g were originally obtained from Vital River Laboratories (VRL, Beijing, China). The rats were housed at the animal facilities of the Institute of Chinese Materia Medica, China Academy of Chinese Medical Sciences, under environmentally controlled conditions (temperature, 22–25 °C; relative humidity, 50%–60%; and day-night light cycle, 12 h–12 h). All experimental procedures were approved by the Animal Ethics Committee of China Academy of Chinese Medical Sciences.

### 3.4. Drug Administration and Chronic Unpredictable Mild Stress (CUMS) Procedure

After seven days of adaptation, the rats were randomly separated into six groups(*n* = 8) including one blank control group, one model group, one positive control group and three treatment groups according to the body weights and behavior scores in open-field experiments. Rats were intragastrically administered 10 mL∙kg^−1^ (rat body weight) of the treatments once a day. Rats in the treatment groups were administered the same amount of solution containing three different concentrations of YHTA (3, 6 and 12 g∙kg^−1^∙d^−1^), respectively. The doses of YHTA refer to crude drug of Corydalis Rhizoma. The positive control group was orally gavaged with venlafaxine (8 mg∙kg^−1^∙d^−1^), while the blank control group and the model group were only administered the same amount of water via gastric intubation. This procedure lasted for 21 days.

The animals, except the blank control group, were individually housed and repeatedly exposed to a set of CUMS as follows [23,47]: (1) damp sawdust for 24 h (200 mL of water per individual cage, which is enough to make the sawdust bedding wet); (2) cage tilted by 45° angle for 24 h; (3) restricted movement for 4 h; (4) swimming in 4 °C cold water for 5min; (5) strange and anomalous items (plastic cup, spoon, broken pieces of cloth); (6) 48 h of food deprivation; (7) 24 h of water deprivation; (8) tail clamp for 1 min, (9) 15 unpredictable shocks (50 mV, one shock/5 s,10 s duration). One stressor was applied per day and the whole stress procedure lasted for 21 days in a completely random order. The blank control group rats were housed six rats per cage and the other groups were housed individually. Water and food were supplied *ad libitum* throughout the study except for the periods of water and food deprivation.

On the last day, rats were tested in the open field after the last stressor, and killed immediately thereafter by decapitation in a separate room. The plasma was obtained by centrifugation at 1150× *g* for 15 min at 4 °C and was frozen immediately at −80 °C prior to NMR spectroscopy analyses and all necessary transport of the samples was carried out on dry ice.

### 3.5. Behavioral Tests

The open-field test was performed in a quiet room on the 21th day as per previous reports. In the open field test, rats were placed at the center of a 100 × 100 cm arena with 40cm-high side walls. The floor was marked with a grid dividing it into 25 equal-size squares. Each animal was placed in the central square and observed for 5 min and each rat was tested once. Scores were calculated by the amount of time it spent on the central square (immobility time) and the number of rearing (defined as standing upright on its hind legs) and the number of ambulations (grid lines it crossed with at least three paws). All these measures were summed to yield the total open field activity. At the same time, the body weight of each rat was recorded.

Sucrose preference test was performed as follows: after a 24h period of water and food deprivation, each rat was placed in an individual metabolic cage in which two bottles containing water and 1% sucrose solution were placed. The ratio of the amount of sucrose solution to that of total solution ingested within 1 h represented the parameter of hedonic behavior.

The significance of variation between groups in data of behavior changes was determined using an independent sample *t*-test by SPSS 11.5 for Windows and the threshold *p* value was set at 0.05 throughout the study. The behavior data of each group was compared with that of the model group and the control group, respectively. The data were presented as mean ± SE.

### 3.6. ^1^H-NMR Method

#### 3.6.1. Sample Preparation and ^1^H-NMR Spectroscopy

After being thawed at room temperature, 400 μL of plasma was taken and mixed with 30 μL 600 mM PB solution (2.2130 g K_2_HPO_4_∙3H_2_O and 0.3782 g NaH_2_PO_4_∙2H_2_O were metered volume to 20mL with deuteriumoxide) and 170 μL deuteriumoxide. Then the samples were centrifuged at 12,000 rpm for 10 min at 4 °C, and the supernatants (500 μL) were collected and placed into 5 mm diameter NMR tubes. NMR analyses were performed at 25 °C and 599.91 Hz using a Varian Unity INOVA 600 NMR spectrometer. One-dimensional spectra were recorded using the Carr-Purcell-Meiboom-Gill sequence with t_m_ fixed at 100 ms. For each sample, 128 transients were collected and with a relaxation delay of 2.1 s and an acquisition time of 1.00 s. The spectral width in each dimension was 8012.8 Hz.

#### 3.6.2. Data Processing of NMR Spectra

NMR spectra were manually corrected for phase and baseline distortion using TopSpin3.0 (Bruker Biospin, Rheinstetten, Germany). The free induction decay was multiplied by an exponential line-broadening function of 0.5 Hz prior to Fourier transformation. All spectra were referenced to the internal lactic acid CH_3_ resonance at 1.33 ppm and the integrals of spectral area include the range 0.5–9.0 ppm, among which residue water signals (δ 5.10–4.66) and urea signals (δ 6.00–5.60) were removed. The spectral area within each bin was integrated and it was segmented into 0.005 ppm chemical shift “bins”. All integral regions were normalized to the total sum of the spectra before pattern recognition. The binned data were subjected to the generalized log transformation (*k* = 0.0025), and the columns were mean centered before multivariate analysis.

#### 3.6.3. Multivariate Statistical Analysis

The mean-centered NMR data were firstly analyzed by PCA using the Simca-P 11.0 software (Umetrics AB, Umea, Sweden). PCA, an unsupervised pattern recognition method, was initially applied to visualize inherent clustering between different groups, which displays the internal structure of datasets in an unbiased way and decreases the dimensionality of data. For regression, PLS-DA was used. A 20-fold cross-validation was employed to obtain Q^2^ and R^2^ values, which represent the predictive ability of the model and the explained variance, respectively. To further validate the quality of the PLS-DA model, permutation tests consisting of a randomly permuting class membership and running 200 iterations were carried out. Next, OPLS-DA was employed to maximize covariance between the measured data (peak intensities in NMR spectra) and the response variable (predictive classifications). OPLS-DA as an extension of PLS-DA featuring an integrated orthogonal signal correction (OSC) can remove variability not relevant to class separation. Both PLS-DA and OPLS-DA were based on unit variance scaling strategy. The metabolites associated with the group separations were indicated by the loadings and coefficients in the coefficient loading plots calculated by back transformation of the loadings. The correlation coefficient of |r| > 0.666 was used as the cutoff value for the statistical significance based on the discrimination significance at the level of *p* = 0.05 and df (degree of freedom) = 7. The coefficients were color-coded (gradient of red colors for positive values and gradient of blue colors for negative values).The presence of colored pixels between specific metabolites represents a correlation (above the cutoff) between these metabolites, which may reflect functional correlations.

## 4. Conclusions

Our results demonstrate that^1^H-NMR-based metabolomics coupled with multivariate statistical analysis is a useful tool for the investigation of TCMs in terms of their therapeutic effects and mechanisms of action involved. The therapeutic efficacy and mechanisms of YHTA on depression were demonstrated by behavioral tests and global plasma metabolite levels. In the study, thirteen plasma metabolites were identified as potential biomarkers of CUMS rats: *myo*-inositol, glycerol, glycine, creatine, glutamine, glutamate, β-glucose, α-glucose, acetoacetate, 3-hydroxybutyrate, leucine, and unsaturated lipids (L7, L9), which are involved in amino acid metabolism, glycometabolism, lipid metabolism and energy metabolism. YHTA plays an anti-depressant role through regulating the above endogenous plasma metabolites. The method used in this study can provide a simple and easy tool for herbal therapeutic evaluation.
